# Current and Emerging Therapies for Eosinophilic Esophagitis (EoE): A Comprehensive Review

**DOI:** 10.3390/pharmaceutics17060753

**Published:** 2025-06-07

**Authors:** Brooke G. Musburger, Maria Gonzalez Echeandia, Elias L. Suskind, David L. Suskind, Hengqi Betty Zheng, Dominique Mark

**Affiliations:** 1 Division of Pediatric Gastroenterology, Seattle Children’s Hospital, Seattle, WA 98105, USA; brooke_g_musburger@rush.edu (B.G.M.); maria.gonzalezecheandia@seattlechildrens.org (M.G.E.); david.suskind@seattlechildrens.org (D.L.S.); betty.zheng@seattlechildrens.org (H.B.Z.); 2 School of Life Sciences, Arizona State University, 427 E Tyler Mall, Tempe, AZ 85281, USA; esuskin1@asu.edu; 3 Department of Pharmacy, Seattle Children’s Hospital, Seattle, WA 98105, USA

**Keywords:** eosinophilic esophagitis, EoE, EGID

## Abstract

Eosinophilic Esophagitis (EoE) is a chronic, immune-mediated disorder that is characterized by symptoms of esophageal dysfunction and the presence of increased eosinophils in the esophageal mucosa. It is becoming increasingly prevalent among children and adults and its pathogenesis arises from the complex interaction of genetic predisposition and environmental triggers, both which contribute to esophageal inflammation. Current societal guidelines recommend the use of proton pump inhibitors, topical steroids, and dietary interventions such as elimination diets as first-line treatments, however, the recent approval of Dupliumab has provided an additional therapeutic avenue. There are a number of investigational biologic agents targeting other immune pathways which are making their way through the pipeline of pharmacologic options in treating this chronic disorder.

## 1. Background

Eosinophilic esophagitis (EoE) is a chronic immune-mediated disorder characterized by symptoms of esophageal dysfunction and the presence of increased eosinophils in the esophageal mucosa. The detection of eosinophils in the esophageal tissue occurs in with the absence of identifiable secondary causes of eosinophilia. The incidence and prevalence of EoE has increased significantly, particularly in Western countries [1], and now, the disease ranks as the most common cause for dysphagia and bolus impaction in children and young adults. It has been identified as the second most common cause of chronic esophagitis, following gastroesophageal reflux disease [1]. Eosinophilic esophagitis (EoE) can be treated using a wide range of therapies, including dietary elimination, acid suppression, steroids, and more recently, biologic medications. In this review, the authors aim to provide an overall review of EoE therapeutics both in use and in clinical development in pediatrics and adults.

## 2. Epidemiology and Clinical Presentation

Eosinophilic esophagitis is becoming increasingly prevalent among both children and adults. The incidence rises during adolescence and peaks in early adulthood [2]. The presentation of EoE can differ based on the patient’s age and symptom severity. In children, common symptoms include failure to thrive, choking, regurgitation, postprandial vomiting, and food refusal. Adolescents and adults often present with retrosternal discomfort, dysphagia (70% of cases), food impaction (33–54% of cases), and persistent dyspepsia, which may respond poorly to proton pump inhibitors (PPIs) [3]. Many patients also develop adaptive eating behaviors, such as intentionally consuming smaller portions, avoiding certain foods, or using sauces and liquids to facilitate the act of swallowing [3]. While symptoms are usually chronic in nature, patients may still experience acute episodes, such as food impaction.

A significant portion of EoE patients (up to 75%) have a personal or family history of allergic conditions, such as allergic rhinoconjunctivitis, eczema, or asthma [3]. Approximately 50% of patients exhibit peripheral eosinophilia or elevated serum IgE levels. Additionally, 75% of patients test positive for at least one food allergen in skin prick tests, with common allergens including dairy, eggs, peanuts, fish, wheat, soy, and aeroallergens like dust mites and pollen. While children with EoE are more likely to have concurrent food allergies, adults are often more sensitive to aeroallergens [3].

## 3. Pathogenesis

**Figure 1:**
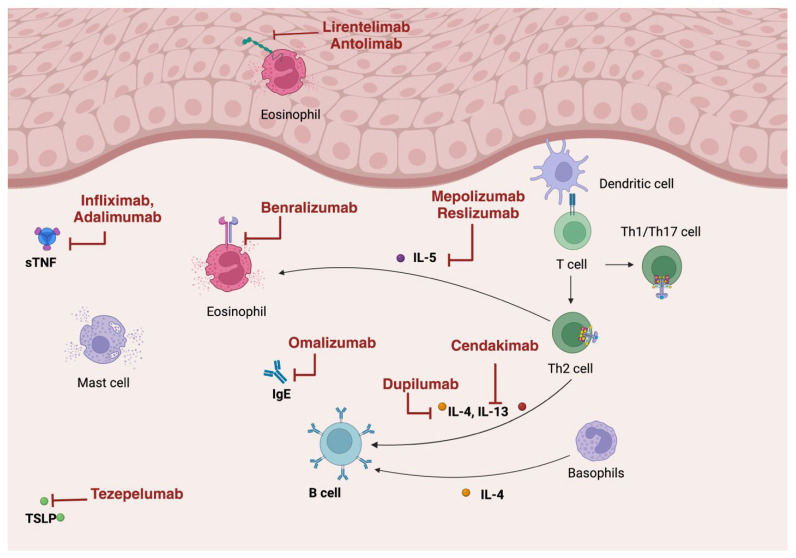
Biologic agent target sites of action.

Eosinophilic esophagitis arises from the complex interaction of genetic predisposition and environmental triggers, which contribute to esophageal inflammation. This condition is characterized by dysfunction of the esophageal epithelium and an abnormal T-helper type 2 (Th2) cell-mediated immune response to environmental allergens (Figure 1). Together, these factors lead to esophageal damage, impaired motility, and long-term complications such as remodeling and fibrosis in the esophagus [4].

### 3.1. Genetic Factors

Eosinophilic esophagitis (EoE) is influenced by genetic predisposition, as evident from an increased risk among first-degree relatives. Genome-wide association studies (GWAS) have identified several genes associated with EoE, primarily affecting epithelial barrier function and Th2-mediated immune responses. Among these, thymic stromal lymphopoietin (TSLP) plays a key role in creating a pro-Th2 immune environment, contributing to EoE development [4]. Additional genes within the epidermal differentiation complex, such as Desmoglein-1 (DSG1), Calpain-14 (CALPN14), and filaggrin (FLG), are implicated in epithelial cell differentiation and barrier function [4]. Notably, FLG, a critical epithelial protein, is downregulated in EoE. Furthermore, serine peptidase inhibitors Kazal type 5 and 7 (SPINK5 and SPINK7) play roles in EoE by disrupting serine protease regulation, leading to increased tissue permeability and an abnormal Th2-mediated immune response [4,5]. The Th2-mediated immune response is driven by cytokines, including IL-4, IL-5, and IL-13. IL-4 facilitates T cell differentiation into Th2 cells and activates B cells. IL-5 promotes eosinophil proliferation, survival, activation, and chemotaxis, and IL-13 leads to multiple effects, such as eotaxin-3 (CCL26) production, eosinophil attraction, and tissue remodeling [4,5]. Tissue remodeling in EoE includes increased collagen deposition, angiogenesis, and epithelial hyperplasia, which are characteristic features of EoE that lead to the progression of the disease [4,5].

The interaction between DSG1 and periostin (POSTN) highlights the link between epithelial barrier dysfunction and the Th2-mediated immune response. Downregulation of DSG1 leads to an increase in POSTN, resulting in the production of TSLP by esophageal epithelial cells. TSLP amplifies the Th2 immune response while also activating mast cells and basophils. Additionally, the STAT6 gene, which is activated by IL-4 and IL-13, encodes a transcription factor that regulates multiple EoE-related genes, further linking cytokine signaling to disease progression [4,5].

### 3.2. Environmental Factors

The rising incidence of EoE underscores the critical role of environmental factors in its development. Environmental allergens with seasonal variations, rural living, and antigen exposure are associated with the onset and exacerbation of the disease.

Atopy, a genetic predisposition to developing allergies, is observed in 75% of EoE patients [4]. Atopic dermatitis (AD), IgE-mediated food allergy, and asthma independently and cumulatively increase the likelihood of an EoE diagnosis. Children with IgE-mediated food allergies, especially those with multiple food allergies, have a significantly higher risk of developing EoE compared to children without food allergies. The risk is further elevated in patients with IgE-mediated food allergies undergoing oral immunotherapy [4]. Additionally, early-life environmental exposures, including maternal fever, pre- and postnatal antibiotic use, proton pump inhibitor (PPI) therapy, and neonatal intensive care unit admission, have been associated with an increased risk of EoE [6]. It is hypothesized that these early exposures may disrupt gut microbiota, subsequently influencing the normal development of the immune system [6].

Interestingly, *Helicobacter pylori* (*H. pylori*) appears to exert a protective effect against esophageal eosinophilia [4]. This protection may be due to *H. pylori* driving a Th1-mediated immune response, which shifts the Th1–Th2 balance and shields against an allergic Th2 response associated with EoE [4].

## 4. Diagnosis

EoE presents with esophageal symptoms such as dysphagia in older children and adults or feeding intolerance and GERD-like symptoms in younger children.

The three primary diagnostic criteria for EoE include the presence of symptoms related to esophageal dysfunction, eosinophil-predominant inflammation, defined as ≥15 eosinophils per high-power field (hpf) in esophageal tissue, and the exclusion of other disorders that may cause esophageal eosinophilia, such as reflux. Biopsy findings showing increased eosinophil counts in the esophagus remain the gold standard for diagnosis. Due to the patchy nature of eosinophilic infiltration in EoE, multiple biopsies (>5) from multiple esophageal levels are required. Histologic features of EoE may also include microabscesses, basal zone hyperplasia, dilated spaces, epithelial changes, dyskeratotic cells, and fibrosis.

Endoscopic evaluation of EoE is associated with various abnormalities, including a narrowed esophagus (9%), longitudinal ridges or furrows (48%), ‘corrugated’ rings or trachealization (44%), strictures (21–40%), Schatzki rings, mucosal tears, and a ‘crepe paper’ effect (59%) caused by mucosal fragility [3]. Additionally, eosinophilic abscesses with white-speckled exudates (27%) may be observed [3]. Adults typically exhibit more subepithelial fibrosis and esophageal narrowing compared to children as fibrosis tends to progress over time [3].

## 5. Societal Guidelines

Current guidelines focus on the diagnosis, standard treatment options, and monitoring of EoE. These guidelines recommend the use of proton pump inhibitors, topical steroids, and dietary interventions such as elimination diets as first-line treatments. Biologic therapies, including anti-IL-5 and anti-IL-13 agents, are newly emerging, with some still in clinical trials due to limited evidence. In 2022, the Food and Drug Administration (FDA) approved the use of dupilumab (Dupixent) for EoE in children who are at least 12 years of age and further expanded approval in early 2024 to children down to at least 1 year of age [7]. Its use has also been described in the European Society of Pediatric Gastroenterology, Hepatology, and Nutrition (ESPGHAN) position statement, with strong recommendations for EoE treatment in children over 1 year of age weighing at least 15 kg with refractory disease [8].

The 2020 guidelines by the American Gastroenterological Association (AGA) and the Joint Task Force on Allergy and Immunology Practice Parameters (JTF) provide key recommendations for managing EoE [9]. For patients with symptomatic eosinophilic esophagitis, proton pump inhibitors (PPIs) are recommended over no treatment. In addition, topical glucocorticosteroids are preferred over no treatment, particularly in children. For patients who achieve remission after short-term topical steroid use, continuing treatment may be advisable due to a fairly low side effect profile [9]. Dietary interventions may take many forms. An elemental formula diet may be considered, though adherence can be challenging. Other options include food elimination diets that range from single food elimination (dairy) to six-food elimination diet (dairy, eggs, wheat, soy, tree nuts and peanuts, fish, and shellfish), which can prove difficult to follow [9]. Allergy testing-based elimination diets have also been recommended, but with caution due to limited accuracy of the testing [9].

Similarly, the 2022 British Guidelines on managing eosinophilic esophagitis consider elimination diets to be effective in achieving histological remission in both adults and children. Among these, the six-food elimination diet was noted to demonstrate higher remission rates compared to the two- or four-food diets; however it is associated with lower compliance and need for endoscopic assessment at each elimination step [2]. While dietary restrictions combined with pharmacological treatments are not recommended, this approach may be considered if drug treatments fail. The British Guidelines do not advise allergy testing for selective dietary restrictions, and exclusive elemental diets are reserved for patients unresponsive to other treatments due to their low compliance rates.

For pharmacologic therapy, the British Guidelines note that proton pump inhibitors (PPIs) are effective in inducing both clinical and histological remission and should be administered twice daily for at least 8–12 weeks before assessing histological response. Those who respond well can maintain remission with continued long-term PPI therapy. Topical steroids are also noted to be effective, but relapses are common after therapy withdrawal; hence, additional pharmacologic options should be explored. Systemic steroids, sodium cromoglycate, montelukast, and antihistamines are not recommended for eosinophilic esophagitis, although they may be useful for coexisting atopic conditions. If symptoms recur during treatment, repeating endoscopy for assessment is advised [2].

In terms of weaker recommendations, immunomodulators such as azathioprine or 6-mercaptopurine, as well as monoclonal antibodies used for inflammatory bowel disease, are not recommended for eosinophilic esophagitis [2].

## 6. Treatment Guidelines for EoE

### 6.1. Dietary Therapies

In 2006, Kagalwalla et al. demonstrated that eliminating common food allergens from the diets of children with EoE induced remission in up to 74% of patients, confirming the role of food allergens in EoE [10]. Dietary regimens have proven to be effective long-term treatments, comparable to topical steroids and PPIs. However, they require regular follow-up endoscopies after each food reintroduction within 6–12 weeks to confirm endoscopic and histologic remission.

Differing approaches exist for dietary interventions, especially when considering that empiric elimination diets exclude common trigger foods such as milk/dairy, wheat/gluten, egg, soy, nuts, and seafood. While a six-food elimination diet achieves higher histologic remission rates than two- or four-food elimination diets, it is associated with lower compliance and a greater number of endoscopies [2]. Thus, a one-food elimination diet (OFED) has gained popularity. Studies consistently identify milk/dairy and wheat as the most common triggers in EoE patients, and up to 70% of patients successfully treated with a TFED (Trial Food Elimination Diet) are found to have a singular trigger, with milk being the most frequent [10]. As a result, multiple studies are re-evaluating the effectiveness of a one-food elimination diet (OFED) that restricts either milk or wheat/gluten exclusively as an acceptable alternative to a six-food elimination diet.

Target elimination diets aim to identify and exclude specific trigger foods based on the results of food allergy tests. While both skin prick tests (SPTs) and atopy patch tests (APTs) have been investigated for this purpose, their effectiveness has been limited [10]. Up to 67% of patients with EoE have IgE-mediated food allergies, and approximately 2% have celiac disease, making overly restrictive diets impractical or unnecessary [10].

### 6.2. Elemental Formulas

Cow’s milk protein (CMP) is the food antigen most frequently associated with EoE in both pediatric and adult patients, identified as a trigger in approximately 3 of 4 EoE patients [11].

Allergy to CMP is a common condition with a wide range of effects, ranging from anaphylaxis to skin conditions and gastrointestinal disturbances, especially in children [11]. Cow’s milk-based extensively hydrolyzed formulas (eHFs) are used to manage CMP allergies due to being well tolerated by most infants and children [11]. Lucendo et al. documented that the majority of adult EoE patients who are triggered by cow’s milk can tolerate an eHF based entirely on CMP. This provides a safe alternative to cow’s milk without risk of EoE recurrence [11].

Markowitz et al. demonstrated that an elemental diet using an amino acid-based elemental formula yielded overwhelmingly positive results, with significant symptom improvement within 10 days, as well as a dramatic reduction in the number of eosinophils infiltrating the esophagus [12]. An ESPGHAN position statement recommends the use of amino-acid formula (AAF) as an option in patients with multiple food allergies, failure to thrive, or those with severe disease who do not respond, or are unable, to follow highly restricted diets as they have shown to be highly effective, but drawbacks include high cost, poor compliance, and palatability.

While exclusive elemental diets are highly effective in managing EoE, their role is limited due to low compliance rates. As such, they are typically reserved for patients refractory to other treatments [2].

### 6.3. Steroids

Topical steroids are effective in inducing clinical and histologic remission in patients with EoE and are typically used in those individuals who have not responded to PPI therapy. The most common topical steroids given for EoE include fluticasone propionate and budesonide. In a double-blind clinical trial from Dellon et al., 111 newly diagnosed adults with EoE were randomized to receive either fluticasone 880 μg swallowed twice daily from a multidose inhaler or oral viscous budesonide 1mg twice daily for 8 weeks [13]. In the budesonide group, peak eosinophil counts declined from 73 to 15 eosinophils/hpf, and in the fluticasone group, from 77 to 21 eosinophils/hpf (*p* = 0.31). Histologic response with reduced eosinophil count was achieved in 71% of the budesonide group and 64% of the fluticasone group [13]. Similarly, in a randomized, double-blind, placebo-controlled trial, 28 adolescent and adult patients were randomized to receive either budesonide at 0.5mg divided twice daily or placebo over a 50-week time period for EoE treatment. Authors found that low-dose budesonide was more effective than placebo for both clinical and histologic remission, and there were no reports of epithelial atrophy [14]. Topical steroids have a relatively low side effect profile and are considered safe, with the most common adverse effect being esophageal candidiasis, occurring in 5–30% of cases [15,16]. Herpes esophagitis has also been reported as a potential complication [17].

Systemic steroids are not currently recommended as first-line therapy for inducing remission in EoE [2]. Available guidelines do not favor their use due to a lack of data and their greater side effect profile (weight gain, hyperphagia, secondary adrenal insufficiency, bone mineral density abnormalities, glaucoma, hyperglycemia, and cushingoid features) when compared to topical steroids [13].

Topical and systemic corticosteroids are similarly effective in reducing both symptoms and esophageal eosinophilic infiltration in EoE. A randomized controlled trial by Schaefer et al. evaluated 80 pediatric EoE patients treated with either topical fluticasone or prednisone [7]. Although patients receiving prednisone showed a greater degree of histologic improvement than those on fluticasone, there was ultimately little difference in symptomatic relief. After 1 month of therapy, 100% of prednisone patients and 97.2% of fluticasone patients achieved symptom resolution. Histologic improvement was observed in 30 of 32 prednisone patients, for whom complete biopsy data were collected, and in 34 of 36 fluticasone patients. Complete histologic remission, defined as normal biopsy results after 1 month of treatment, was achieved in 26 of 32 (81%) prednisone patients and 18 of 36 (50%) fluticasone patients [7].

### 6.4. Proton Pump Inhibitors

Proton pump inhibitors (PPIs) are considered a traditional first-line treatment for EoE, offering similar efficacy as topical steroids and dietary therapy [4]. PPIs have been shown to induce both histologic and symptomatic improvement in patients with EoE. A 2020 review from the American Gastroenterological Association Institute and Joint Task Force on Allergy and Immunology Practice Parameters evaluated 23 observational studies with 1051 total patients, and found that PPI therapy was associated with a histologic response (defined as <15 eosinophils/hpf) in 41.7% of patients compared to 13.3% in the placebo group [18]. A 2016 meta-analysis of 33 studies (including 11 prospective), with 619 patients suffering from EoE (188 children and 431 adults) indicated that PPI therapy resulted in symptomatic improvement for 60.8% of patients and histologic remission in 50.5% [19]. The effectiveness of this drug class may be reduced in patients who are nonresponsive to dietary therapy or topical steroids or in those with fibrostenotic phenotype [20].

Commonly prescribed PPIs for EoE include Omeprazole, Lansoprazole, Esomeprazole, and Pantoprazole [4]. One approach for PPI initiation recommends starting once daily dosing for 8 weeks, and if no symptomatic improvement is observed after 4 weeks, the dose may be increased to twice daily [21]. After 8 weeks of treatment, an EGD is performed to evaluate endoscopic and histologic improvement. If improvement is observed, PPI therapy is continued at the lowest effective dose to maintain symptom control. If no improvement occurs, alternative treatments such as dietary therapy and topical steroids are considered [21]. British guidelines recommend initiating PPI therapy at a twice-daily frequency as higher doses have been associated with improved clinicopathological response rates when compared to standard or low-dose regimens. Authors indicate that agents and doses do vary widely in the literature for the treatment of EoE [2].

Long-term use of proton pump inhibitors (PPIs) has been associated with several safety concerns, including acute and chronic kidney disease, dementia, bone fracture and osteoporosis, myocardial infarction, Clostridium difficile infection and microscopic colitis, pneumonia, micronutrient deficiency anemia, hepatic encephalopathy, and fundic gland polyps [22]. However, the absolute increased risk of these adverse effects is generally small (between 0.03% and 1.5%), and the evidence linking PPIs to these outcomes is insufficient to establish definitive causation [23].

## 7. Newer Agents in Treatment for EoE

Biologic therapies such as the newly approved anti-IL-4/13 antibody dupilumab and other anti-cytokine and protein targeting antibodies, have been changing the paradigm of EoE treatment over the last few years. Many therapeutics are still in clinical trials. Here, the authors aim to describe these therapeutics in a review of the literature (Figure 1, Table 1 and Table 2, and Supplementary Tables S1–S5).

### 7.1. IL-4/IL-13 Antagonists

#### 7.1.1. Dupilumab

In 2022, dupilumab became the first FDA-approved drug for the treatment of EoE due to its significant impact on histologic outcomes and the Dysphagia Symptom Questionnaire. Dupilumab is a monoclonal antibody that targets the alpha subunit of the interleukin-4 receptor and blocks both the IL-4 and IL-13 signaling pathways by preventing dimerization with IL-13Rα1. The pharmacokinetics of dupilumab are unique, with a target-mediated phase and terminal half-life that cannot be calculated as the drug’s instantaneous half-life decreases over time to values approaching zero [24]. Dupilumab’s elimination follows target-mediated clearance and nonlinear kinetics, with internalization and catabolism into small peptides and amino acids by the reticuloendothelial system [24]. Peak drug concentrations are reached 3–7 days after a single subcutaneous injection, with clinical effects typically seen within 2–4 weeks. Steady-state concentrations are obtained within 12–24 weeks, depending on the frequency of administration [25] (Table 1).

Monitoring for hypersensitivity reactions, including elevated blood pressure, heart rate, and rare ocular side effects after injection, is recommended. Common adverse drug reactions include injection site reactions, arthralgia, herpes viral infections, and upper respiratory tract infections. In addition, pediatric patients <12 years have a potential increased risk for parasitic (helminth) infection [42] (Table 2).

In a phase 3 clinical trial by Chehade et al., patients 1 to 11 years of age with active eosinophilic esophagitis and no prior response to PPI therapy were randomly assigned to either a high-dose or low-dose dupilumab group, or to one of two placebo groups, for 16 weeks (four groups total, with a 2:2:1:1 ratio) in Part A of the study (see Table S1 for group-specific dosing in regards to high-dose and low-dose administration). In Part B, patients in the high-dose and low-dose dupilumab groups continued their respective regimens, while those in the placebo groups were switched to either high- or low-dose dupilumab for 36 weeks. Part C of the study is an ongoing 108-week open-label extension, with results yet to be released. In Part A, histologic remission (as defined by a peak eosinophil count ≤ 6 per hpf or ≤20 per square mm) was achieved by 68% of patients in the high-dose group, 58% of patients in the low-dose group, and 3% of patients in the placebo group. At the conclusion of Part B, improvements in histologic, endoscopic, and transcriptomic measures were generally similar to those observed in Part A for patients receiving dupilumab compared to baseline [43] (Table S1).

When considering available therapeutic options for the management of EoE, both patient and provider perspectives should be taken into consideration when discussing the feasibility of medication attainment. Monoclonal antibody therapy is notably expensive and may pose challenges for patients and caregivers in gaining insurance approval for use. In a 2023 study by Nguyen et al., the authors aimed to explore physician and patient perspectives on initiating dupilumab for EoE [44]. The study utilized a retrospective chart review to assess prescribing practices from both the physician’s and patient/family perspectives [34]. From the physicians’ perspective, the main reasons for initiating dupilumab were a lack of response to topical corticosteroids (52%), patient nonadherence (28%), adverse effects of steroids (10%), or treating multiple atopic diseases (5%). From the patients’ point of view, the primary reasons for dupilumab therapy included a lack of response to topical corticosteroids (27%), nonadherence (27%), concern about adverse effects of topical corticosteroids (7%), and treatment of multiple atopic diseases (33%). Almost all patients (98%) required prior authorization, with 17% needing an appeal letter and 2% requiring a peer-to-peer review. Overall, the study highlighted that physicians primarily prescribe dupilumab for nonresponse to topical corticosteroids, and almost all patients faced delays in treatment due to challenges with insurance coverage [44] (Table S1).

A study by Dellon et al. evaluated the efficacy and safety of dupilumab in adults and adolescents (≥12 years old) with EoE in a three-part, randomized, phase 3 trial [45]. The inclusion criteria required patients to have a peak eosinophil count ≥ 15/hpf despite 8 weeks of high-dose PPI therapy, and a Dysphagia Symptom Questionnaire (DSQ) score of ≥10. In Part A, 42 patients received 300 mg dupilumab weekly and 39 patients received a placebo for 24 weeks. In Part B, 80 patients received 300 mg of dupilumab weekly, 81 received 300 mg of dupilumab every 2 weeks, and 79 patients received a placebo weekly for 24 weeks. The patients who received dupilumab every 2 weeks in Parts B and C also received a placebo, which was provided in an alternating schedule with the active drug. Eligible patients in remission from Part A and Part B continued the trial in Part C. In Part C, 77 patients from Part A continued 300 mg weekly dupilumab for an additional 28 weeks, totaling 52 weeks in duration (40 patients who had been receiving 300 mg weekly dupilumab in Part A continued the same regimen, while 37 patients switched from a placebo to 300mg weekly dupilumab) [45]. Part C, which includes eligible patients from Part B, is still ongoing. In Part A, histologic remission was achieved in 25/42 (60%) patients who received weekly dupilumab and in 2/39(5%) patients who received the placebo. In Part B, histologic remission was achieved in 47/80 (59%) patients receiving weekly dupilumab, in 49/81 patients (60%) receiving dupilumab every two weeks, and in 5/79 (6%) patients with the placebo. Among patients from Part A who continued weekly dupilumab in Part C, the effects were sustained by the end of week 52, with histologic remission observed in 19/34 (56%) of these patients. Patients who initially received placebo in Part A and later started on dupilumab in Part C showed treatment effects at week 52, similar to those seen in patients who started weekly dupilumab in Part A, with histologic remission achieved in 18/30 (60%) patients by the end of week 52 [45].

Patients in all three study groups who received weekly dupilumab demonstrated a significant reduction in Dysphagia Symptom Questionnaire (DSQ) scores compared to patients given the placebo in their respective groups. However, in Part B, the reduction in DSQ scores did not differ significantly between the patients who received dupilumab every two weeks and those who received placebo [45].

In all three trial groups, the incidence of adverse events ranged from 60 to 86%, with injection site reactions being the most commonly reported among those who received dupilumab [45]. Serious adverse events, including site reactions and infections, were reported in 9 patients from Part A and B: 7 patients on weekly dupilumab, 1 on dupilumab every other week, and 1 on the placebo, as well as 1 patient who received a placebo in Part A and then dupilumab in Part C. No deaths occurred. Overall, this phase 3 trial demonstrated that weekly dupilumab significantly improved histologic outcomes and alleviated symptoms of EoE in adults and adolescents [45] (Table S1).

#### 7.1.2. Cendakimab

Interleukin-13 (IL-13) is a cytokine that plays a critical role in the pathogenesis of eosinophilic esophagitis by inducing chemokines that activate eosinophils, promoting tissue remodeling with collagen deposition and angiogenesis, disrupting the epithelial barrier and stimulating eosinophil survival and activation. Cendakimab is a monoclonal antibody targeting IL-13, which reduces histologic and endoscopic activity in EoE patients by blocking the interaction of IL-13 with the IL-13Rα1 and the IL-13Rα2 receptors [46].

Hirano et al. conducted a multicenter, double-blind trial involving 99 adults with active EoE, defined by dysphagia symptoms lasting at least 4 days within the previous 2-week period and histological evidence of EoE based on peak eosinophil count of >15/hpf at baseline [47]. Patients were randomized to receive weekly subcutaneous cendakimab, with 31 patients receiving 180mg weekly, 34 patients receiving 360 mg weekly, and 34 patients receiving a weekly placebo for 16 weeks. In both active dosing arms, significantly more patients achieved a reduction in mean esophageal eosinophil counts compared to the placebo, which showed no change. Similar improvements were observed for peak eosinophil counts, endoscopic severity (measured by the EoE Endoscopic Reference Score (EREFS)), and histologic severity (measured by the EoE Histologic Scoring System (HSS)). Fifty percent of patients receiving 180mg and 360mg weekly achieved a peak eosinophil count of <15 peak eosinophil/hpf, compared to 0% in the placebo group. Additionally, 25% of patients in the 180mg (*p* = 0.0027) and 20% of patients in the 360mg (*p* < 0.0079) group achieved peak eosinophil counts of <6 peak eosinophil/hpf [47] (Table S1).

Dellon et al. conducted a long-term extension (LTE) of the phase 2 study by Hirano et al. [47,48], enrolling 66 patients from the original phase 2 trial into a 52-week LTE. All patients were given 360 mg of cendakimab weekly [48]. Patients who had received a placebo during the double-blinded portion of the study and were switched to 360 mg weekly cendakimab during the LTE showed clinical response by the end of week 12. By week 12 of the LTE, no significant differences were observed in mean and peak esophageal eosinophil counts, total EoE Endoscopic Reference Scores, and EoE Histologic Scoring System grade and stage scores between patients who initially received the placebo and those who had received either dose of active drug. Symptom remission was defined as an EoE activity index score of ≤20. In patients who switched from the placebo to Cendakimab, the remission rate increased from 14% at LTE entry to 67% at week 52. In patients who had received cendakimab throughout (regardless of the starting dose), the remission rate increased from 30% to 54%. Among 28 patients who did not achieve a histologic response to cendakimab during the double-blind induction phase, 10 (36%) achieved a response during the LTE [48] (Table S1).

### 7.2. IL-5 Antagonists

#### 7.2.1. Mepolizumab

Interleukin-5 is a potent proinflammatory T_H_2 cytokine implicated in the pathogenesis of EoE [49]. IL-5 regulates eosinophil function by inducing proliferation, release from the bone marrow into peripheral circulation, maturation, activation, and survival [49]. Mepolizumab is an anti-IL-5 monoclonal antibody that is effective in managing EoE. It has a half-life of 16–22 days, bioavailability of 80%, and undergoes proteolytic degradation throughout the body, not restricted solely to hepatic tissue (Table 1).

Drug monitoring for mepolizumab includes obtaining absolute eosinophil count for hypereosinophilic syndrome and monitoring for potential side effects, including vomiting, diarrhea, upper abdominal pain, injection site reaction, flu-like symptoms, and fatigue (Table 2).

Assa’ad et al. conducted a multicenter, double-blinded, randomized, prospective study involving 59 children with EoE [50]. The diagnosis of EoE was defined as a baseline peak count of esophageal eosinophils of ≥20 per high-power field (hpf). All patients received an infusion every 4 weeks for 12 weeks, totaling 3 infusions. Nineteen patients were dosed at 0.55 mg/kg, 20 patients at 2.5 mg/kg, and 20 patients at 10 mg/kg of mepolizumab, with no placebo group included. Baseline peak esophageal eosinophil counts were 122.5 ± 8.78 per hpf, and mean counts were 39.1 ± 3.63 per hpf. One month after the third infusion, peak eosinophil counts were reduced to <5 per hpf in 5 of 57 patients (8.8%) and <20 per hpf in 18 of 57 patients (31.6%). A mean eosinophil count <20 per hpf was observed in 51 of 57 (89.5%) patients. No significant differences were observed between the different mepolizumab dosing groups. Overall, mepolizumab was found to significantly reduce esophageal eosinophilic inflammation in children with EoE [50] (Table S2).

Otani et al. conducted a sub-analysis of the Assa’ad et al. study [51] examining the tissue samples from 43 of the original 57 patients. Forty percent of patients responded to mepolizumab therapy (defined as having <15 eosinophils/hpf), and 77% of all patients showed a decrease in mast cell numbers. In mepolizumab responders, epithelial mast cell counts decreased from 62 to 19/hpf (*p* < 0.001), and were significantly lower than in non-responders (*p* < 0.05). Mast cells and eosinophils found in couplets before treatment were significantly decreased only in responders after mepolizumab therapy (*p* < 0.001). Additionally, the mast cell growth factor IL-9, predominantly found in esophageal eosinophils, decreased from 102 to 71 per hpf (*p* < 0.001) following treatment. This study demonstrated that pediatric patients with EoE experienced a significant reduction in mast cells, IL-9^+^ cells, and mast cell–eosinophil couplets in the esophageal epithelium after receiving mepolizumab [51] (Table S2).

Dellon et al. conducted a multicenter, randomized, double-blind, placebo-controlled trial to assess whether mepolizumab was more effective than placebo for improving symptoms of dysphagia in patients with EoE who were unresponsive to PPI [35]. In Part 1, patients aged 16–75 with EoE and dysphagia symptoms (measured by EoE Symptom Activity Index [EEsAI]) were randomized 1:1 to receive either mepolizumab 300 mg or a placebo once monthly for three months. In Part 2, patients who received mepolizumab in Part 1 were continued on 300 mg mepolizumab for an additional 3 months, while patients who received a placebo in Part 1 were switched to 100 mg mepolizumab monthly. At the conclusion of Part 1, EEsAI decreased by 15.4 ± 18.1 points with mepolizumab and 8.3 ± 18.0 points with placebo (*p* = 0.14). At the conclusion of Part 2, EEsAI decreased by 18.3 ± 18.1 points for the mepolizumab/mepolizumab group and 18.6 ± 19.2 for the placebo/mepolizumab group (*p* = 0.85). Overall, mepolizumab did not significantly reduce dysphagia symptoms [35] (Table S2).

Straumann et al. conducted a randomized, double-blind, placebo-controlled study to investigate the pharmacodynamic effect of mepolizumab in patients with EoE [52]. Eleven adult patients with active EoE, defined by >20 peak eosinophil number/hpf and symptoms of dysphagia, were randomized to receive either 750 mg mepolizumab (n = 5) or a placebo (n = 6). Patients received two intravenous doses, 1 week apart. After 8 weeks, patients were reassessed, and those who had not achieved complete remission (defined as <5 peak eosinophil number/hpf) were given two additional doses, 4 weeks apart, with the dosage increased to 1500 mg. Patients initially receiving a placebo were continued on a placebo. By week 4, the mepolizumab group showed a 54% reduction in eosinophil count compared to 5% in the placebo group. No further reduction in eosinophil count was seen in either group for the remainder of the study. While mepolizumab significantly reduced eosinophil numbers and reversed changes in molecules associated with esophageal remodeling, only limited symptomatic improvement was noted [52] (Table S2).

#### 7.2.2. Reslizumab

Similar to mepolizumab, reslizumab is a monoclonal antibody targeting interleukin-5, a proinflammatory cytokine that plays a central role in the maturation, recruitment, and activation of eosinophils, as well as their trafficking to the esophagus [36] (Table 1). Baseline laboratory tests, including CBC with differential, should be performed after initiation of reslizumab. Patients should be closely monitored for hypersensitivity reactions, signs of infection, and symptoms such as cough, nasal congestion, headache, constipation, and upper respiratory infection [37] (Table 2).

Markowitz et al. assessed the long-term safety and efficacy of reslizumab in a study with 12 patients [36]. These patients were initially enrolled in a 3-month randomized control study, with 8 participants continuing into an expanded access program after the RCT, which included open-label extension (OLE) and compassionate use (CU) stages. Three patients remained on reslizumab for the entire 9-year study period. During the RCT, patients received reslizumab at 1, 2, or 3 mg/kg (or placebo) every 4 weeks. In the 3.5-year OLE phase, dosing was 1–3 mg/kg (or placebo) every 4 weeks. For the 4 patients entering the CU phase, the dose was set at 2mg/kg every 4 weeks for 5.5 years. No patients experienced vomiting during the OLE or CU phases. A total of 92% of patients achieved a reduction in eosinophil count to <5/hpf (*p* < 0.001). In the three patients who remained on reslizumab for the full 9-year study period, no eosinophils were found in esophageal biopsies at the study’s conclusion. Reslizumab was generally well tolerated with no serious adverse drug reactions reported. Five patients reported mild adverse drug reactions, most commonly cough and nasal congestion, all of which resolved by the end of treatment. Overall, reslizumab was demonstrated to be safe, well-tolerated, and effective as a long-term treatment for children and adolescents with EoE [36] (Table S2).

Spergel et al. investigated the effects of reslizumab in children and adolescents with EoE who had moderate or severe symptom severity scores, esophageal biopsy with ≥24 intraepithelial eosinophils/hpf, and who were non-responsive to PPIs [53]. A total of 226 patients were randomized to receive infusions of 1, 2, or 3 mg/kg reslizumab or a placebo at weeks 0, 4, 8, and 12. Median reductions in peak esophageal eosinophil counts from baseline to the end of therapy were 59%, 67%, 64%, and 24% in the 1, 2, and 3 mg/kg reslizumab (all *p* < 0.001) and placebo groups, respectively. Improvements in physician global assessment were seen in all groups, including placebo, with no statistical differences seen between reslizumab and placebo groups. Reslizumab was generally well tolerated, with headache and cough being the most commonly reported adverse effects. Despite reductions in eosinophil counts, no significant improvements in quality of life or EoE symptoms were observed [53] (Table S2).

#### 7.2.3. Benralizumab

Benralizumab is an anti–IL–5 receptor α monoclonal antibody that achieves near-complete eosinophil depletion through antibody-dependent cell-mediated cytotoxicity [46]. It is currently used as an add-on therapy for patients 12 years and older with severe eosinophilic asthma. Patients should be monitored for anaphylaxis/hypersensitivity reactions during and after administration, as well as any signs of infection [54].

Rothenberg et al. conducted a phase 3, multicenter, double-blind, randomized, placebo-controlled trial involving adolescent and adult patients with symptomatic and histologically active eosinophilic esophagitis (defined by ≥15 EOS/hpf) [54]. A total of 211 patients were randomized, with 104 receiving benralizumab every 4 weeks and 107 receiving a placebo every 4 weeks. The study included four phases, a 2-to-8-week run-in period, a 24-week double-blind treatment period, a 28-week open-label benralizumab treatment period, and an optional open-label extension treatment period. By the end of 4 weeks, benralizumab significantly reduced esophageal eosinophil counts, maintaining this reduction through the period of 24 and 52 weeks. However, there was no improvement in dysphagia symptoms or in endoscopic findings compared to the placebo. Pathologic examination of esophageal biopsies showed that, despite a decrease in eosinophil count in patients who received benralizumab, there was no change in basal zone hyperplasia or other EoE-related epithelial features, indicating continued disease activity. On endoscopy, no improvement in the severity of edema, furrowing, rings, or strictures was observed in patients who received benralizumab. Similar to other anti-IL-5 biologics, like mepolizumab and reslizumab, this trial highlighted the disconnect between histologic and symptomatic responses in patients treated with benralizumab [54] (Table S2).

### 7.3. Anti-IgE-Omalizumab

In eosinophilic esophagitis (EoE), the immune response is primarily driven by Th2 cytokines, particularly interleukins IL-4, IL-5, and IL-13. IL-4, which is produced by Th2 cells, natural killer (NK) cells, and basophils in response to TSLP, promotes the differentiation of naïve T cells into Th2 cells and stimulates B cells, leading to the production of IgE [55]. Additionally, there is an increased presence and degranulation of mast cells in the esophageal epithelium, suggesting that immediate hypersensitivity mediated by immunoglobulin E (IgE) plays a role in the pathogenesis of EoE [55].

Table S4 summarizes several trials of omalizumab, a biologic agent aimed at reducing IgE levels in patients with EoE. Clayton et al. conducted a prospective, randomized, placebo-controlled trial to assess whether EoE is an IgE-mediated condition. The study included only adult patients over 18 years of age with active EoE (eosinophil ≥ 15/hpf) while on maximum PPI therapy. Patients were randomized to receive either OMAL or a placebo and followed for 16 weeks. Dosing was based on body weight and IgE serum levels. The trial found no significant differences in clinical symptoms or eosinophil count when comparing OMAL groups to placebo [56] (Table S4).

Alternatively, Loizou et al. conducted an open-label, single-center study evaluating the role of OMAL in reducing esophageal tissue inflammation. The study included 15 patients, aged 12–75, with atopy, EoE, and elevated serum IgE (30–700 IU/mL), and those who had failed elimination diets or steroid treatments. Dosing was calculated based on weight (mg/kg/dose) per IgE unit/mL and was administered every 2 or 4 weeks, depending on the dosing of OMAL for allergic asthma, for up to 12 weeks of treatment. Approximately 33% of patients achieved full histologic remission (defined as a peak eosinophil < 15/hpf and endoscopic remission of disease), and 7 out of 15 reported improvements in their symptom score following OMAL treatment. No symptomatic improvement was observed in patients who did not experience histologic improvement. In the pediatric population, 4 out of 11 participants achieved full remission. No serious adverse reactions were reported [41] (Table S4).

Omalizumab has been associated with side effects such as leg pain, fatigue, dizziness, arthralgia, pruritis, and dermatitis in asthma patients. In chronic idiopathic urticaria, common adverse reactions include pharyngitis, nausea, sinusitis, upper respiratory tract infection, arthralgia, headache, and cough. In EoE patients, OMAL is generally well tolerated, with no serious adverse events reported, and side effects similar to those observed in other patient populations [41] (Table 2).

### 7.4. Anti-TNFα

Lastly, in addition to the upregulation of TH2 cytokines, TNF-α is also highly expressed by esophageal epithelial cells in patients with active EoE [38]. Straumann et al. investigated the effect of infliximab, an anti-TNFα, on eosinophilic infiltration and EoE symptoms. The study followed three adult patients with steroid-refractory EoE with active disease treated with infliximab 5 mg/kg/dose IV at weeks 0 and 2. Two of the three patients reported improvement in symptoms as well as a decrease in eosinophils on histology, while one patient reported worsening symptoms and an increase in EOS compared to baseline. Overall, no significant improvement was seen in this patient group [38] (Table S5).

### 7.5. Investigational Therapies

In addition to the established treatments, there are several other therapies currently under investigation for EoE. Researchers are exploring new approaches and potential medications to expand the available treatment options. These therapies aim to target various mechanisms involved in the disease, with the hope of improving patient outcomes and offering more effective solutions for those who do not respond well to current treatments.

Siglec-8 is an inhibitory receptor that is found selectively on human mast cells and eosinophils. When a monoclonal antibody (mAb) binds to Siglec-8, it has been shown to induce the death of cytokine-primed eosinophils and inhibit IgE-mediated mast cell activation [57]. Dellon et al. are investigating lirentelimab, an anti-Siglec-8, in a phase 2/3 clinical trial. This randomized, double-blind, placebo-controlled study is evaluating patients 12 years or older with dysphagia and increased eosinophils (≥15 eosinophils/hpf on esophageal biopsy). The trial includes a high-dose (1 mg/kg/dose for 1 dose followed by 3 mg/kg/dose for 5 doses) and low-dose (1 mg/kg for 6 doses) groups as well as a placebo group. Histological response, defined as a peak eosinophil count reduction to ≤6 eosinophils/hpf was achieved in 88% of patients in the high-dose group and 92% in the low-dose group, compared to 11% in the placebo group (*p* < 0.0001). In the adolescent group (ages 12 and 17 years), 94% of patients in both the high-dose and low-dose groups met the primary endpoint, compared to only 6% in the placebo group. Additionally, changes in the DSQ were measured at baseline and at week 24. For adolescent patients, the high-dose group showed a change of 18.4 points, the low-dose group had a 16.4-point change, and the placebo group had an 8.9-point change. The main adverse reactions were infusion-related reactions and headaches, with only 3 patients reporting serious ADR (details not specified) [58] (Table S4).

Thymic stromal lymphopoietin (TSLP) is an epithelial cell-derived cytokine that plays a central role in driving type 2 inflammation through both innate and acquired immune pathways [59]. Tezepelumab (TEZ) is a human IgG2 monoclonal antibody that blocks the binding of TSLP to its receptor. It is currently approved for the treatment of asthma [59]. AstraZeneca is conducting a randomized, double-blind, parallel-group, placebo-controlled phase 3 study to assess the efficacy and safety of TEZ in EoE. The study includes pediatric patients over 12 years of age (12–80 years old) and at least 40 kg with a confirmed diagnosis of EoE and no other associated gastrointestinal disorders. Participants are randomized to receive high-dose TEZ, low-dose TEZ, or a placebo and are followed for 52 weeks. The study aims to evaluate histologic response (defined as ≤6 EOS/hpf) and changes in DSQ. Results are not yet available [60] (Table S3).

Additional studies are underway for the adult population, including the evaluation of Zemaira (ZEM), an alpha 1-proteinase inhibitor (Alpha-1 Trypsin Inhibitor) therapy, which is thought to help reverse inflammation in the esophagus. The study is evaluating patients 18 years and older with known active EoE, moderate to severe symptoms, and a minimum of 8 weeks of PPI or topical steroid treatment. Patients will receive weekly infusions of 120 mg/kg/dose for 12 weeks. The study will assess changes in A1AT esophageal concentrations at 12 weeks, as well as absolute change in baseline serine protease activity by the end of the treatment period at 12 weeks [61] (Table S3).

Etrasimod is a sphingosine-1-phosphate (S1P) receptor modulator that works by reducing circulating lymphocytes in the blood, which helps decrease inflammation and tissue damage [62]. It is currently approved for use in multiple sclerosis and inflammatory bowel disease in adults. Dellon et al. have developed a phase 2 randomized, double-blind, placebo-controlled study to evaluate the use of Etrasimod in EoE on patients aged 18–65 with a diagnosis of EoE with episodes of dysphagia. Participants are randomized to receive either 1 mg daily, 2 mg daily, or a placebo. The study assesses the percent change in peak eosinophil count from baseline to week 16 along with changes in endoscopic findings (EoE HSS, EREFS), patient global impression of severity (PGIS), and DSQ score. Patients with higher dosing (2 mg) experienced a 46.1% reduction in eosinophil count from baseline at week 16, while the low-dose group showed a 32.5% decrease and the placebo group had only a 7% change. Additionally, the high-dose group demonstrated improvement in EoE-HSS and PGIS scores [63] (Table S3).

## 8. Future Directions

Biologic agents such as dupilumab have traditionally been considered for treatment-refractory EoE after the failure of first-line therapies, such as corticosteroids and PPIs. With recent FDA approval of dupilumab for EoE and continued emergence of data showing positive patient response, it is conceivable that future treatment plans could utilize this biologic agent as a first-line therapy in certain cases. Furthermore, incorporating dupilumab earlier in a patient’s treatment may prove beneficial for individuals affected by multiple comorbid atopic conditions, such as atopic dermatitis, asthma, and chronic rhinosinusitis with nasal polyps. Future studies should examine the efficacy of dupilumab for EoE in patients with other atopic comorbidities to understand the therapy’s impact on overall clinical improvement and the economic benefit of addressing multiple disease processes. There are multiple investigational therapies coming through the pipeline, and future studies will be needed to understand the impact of these therapies on long-term remission rates and safety data.

## 9. Conclusions

The landscape of EoE treatment is evolving with the introduction of FDA-approved dupilumab. Other newer agents are under investigation, and therapeutic choices will expand over the next few years. Here, we hope to aid clinicians in their understanding of EoE through our description of EoE and expand on available and future treatment options for this disease process.

## Figures and Tables

**Table 1:** Pharmacokinetics and pharmacodynamics of biologic agents used to treat eosinophilic esophagitis (EoE).

Agent FDA Approval for EoE	MOA	Half-Life (Days) and Metabolism	Bioavailability	Time to Peak/Time to Clinical Response
Dupilumab [24,25,26] FDA Approved for EoE	Anti-IL-4/ IL-13	Half-life: N/A (instantaneous half-life decreases over time to values close to zero; terminal half-life cannot be calculated)	61–64%	3–7 days after injection for time to peak/2–4 weeks to see clinical effects
		Catabolism, degradation into small peptides and amino acids		Steady state concentrations reached by 12–24 weeks, depending on the frequency of administration
Mepolizumab [27] Investigational	Anti-IL-5	Half-life: 16–22 days	80%	N/A
		Proteolytic degradation distributed in the body, not restricted to hepatic tissue		
Infliximab [28] Investigational	Anti-TNF	Half-life-7–12 days	N/A	2–8 weeks for clinical response (as reported in the IBD population); 8–10 weeks in patients with psoriasis
Reslizumab [29,30,31,32] Investigational	Anti-IL-5	Half-life: 24 days	N/A	N/A
Omalizumab [33,34] Investigational	Anti-IgE	Half-life: 26 days	62%	Peak concentration reached 7–8 days after drug administration
		Degradation of IgG and omalizumab:IgE complexes by reticuloendothelial system		12–16 weeks for the response to therapy to be seen

MOA: mechanism of action; N/A: not applicable; IBD: inflammatory bowel disease; TNF: tumor necrosis factor.

**Table 2:** Baseline screening and monitoring for pharmacologic therapies used to treat EoE.

Medication	Drug Class/MOA	Baseline Labs	Monitoring	Common ADRs/BBW
Dupilumab [26]	Anti-IL-4/IL-13	N/A	Signs/symptoms of hypersensitivity reactions; BP, HR, oxygen saturation 15 min after injection	Injection site reactions, arthralgia, herpes viral infections, and upper respiratory tract infections
Mepolizumab [35]	Anti-IL-5	N/A	Hypereosinophilic syndrome (check absolute eosinophil count)	Vomiting, diarrhea, upper abdominal pain, injection site reaction, flu-like symptoms, fatigue
Reslizumab [31,32,36,37]	Anti-IL-5	CBC with differential	Signs/symptoms of hypersensitivity reactions; signs of infection, periodic monitoring of CBC	Cough, nasal congestion, headache, constipation, upper respiratory infection
Infliximab [28,38]	Anti-TNF	CBC with differential, HBV, TB	CBC every 6-12 months, LFTs, HBV reactivation	Infection (upper respiratory, pharyngitis), infusion-related reactions, abdominal pain, headache BBW: malignancy (hepatosplenic T-cell lymphoma), infection (fungal, viral), TB
Omalizumab [33,34,39,40,41]	Anti-IgE	Serum IgE level (mainly reported in the asthmatic population)	Signs of infection	In asthma patients: leg pain, fatigue, dizziness, arthralgia, pruritis, dermatitis In chronic idiopathic urticaria patients: pharyngitis, nausea, sinusitis, upper respiratory tract infection, arthralgia, headache, cough In EoE patients: generally well tolerated with no serious adverse events reported, similar ADRs reported as with other patient populations

N/A: not applicable; MOA: mechanism of action; ADR: adverse drug reaction; LFTs: liver function tests; CBC: complete blood count; TB: tuberculosis; HBV: hepatitis B virus; BP: blood pressure; HR: heart rate; BBW: black box warning.

## Data Availability

Not applicable.

## Supplementary Materials

The following supporting information can be downloaded at: https://www.mdpi.com/article/10.3390/pharmaceutics17060753/s1, Table S1: Il-4/IL-13 Antagonists-Dupilumab and Cendakimab Published Data (Pediatric and Adult); Table S2: IL-5 Antagonists-Mepolizumab, Reslizumab, and Benralizumab for EoE-Published Data (Pediatric and Adult); Table S3: Investigational Therapies (Pediatric and Adult); Table S4: Anti-IgE-Omalizumab for EoE-Published Data (Pediatric and Adult); Table S5: Anti-TNFα-Infliximab (Adult only). References [18,35,36,38,39,40,41,43,44,45,47,48,50,51,52,53,54,56,58,60,61,63,64,65,66] are cited in the supplementary materials.
