# Supplementary material for: Current and Emerging Therapies for Eosinophilic Esophagitis (EoE): A Comprehensive Review
Source: Pharmaceutics. 2025 Jun 7;17(6):753. doi: 10.3390/pharmaceutics17060753 (PMC12196940; doi:10.3390/pharmaceutics17060753)
Supplement: Supplementary file 1 [file pharmaceutics-17-00753-s001.zip › pharmaceutics-3582793-supplementary.pdf]

Supplement S1: Tables S1–S5

Table S1. Il-4/IL-13 Antagonists-Dupilumab and Cendakimab Published Data (Pediatric and Adult)

Study and Design	Population and Study Objectives	Endpoints/Outcomes	Results	Final Assessment and Pertinent Commentary
Chehade M et al., 2024 [43]<br><br>Dupilumab<br>*Pediatric study*<br><br>**FDA Approval:** Dupilumab is FDA approved for treatment of EoE in children 1-11 years of age	Phase 3 trial-patients: patients 1-11 yrs of age with active EoE as defined by:<br>-≥ 15 EOS count per hpf, clinical hx of symptoms, peak intraepithelial count of > 15/hpf<br>-No response to PPI therapy<br><br>Part A: (weeks 1-16), randomized, double-blind, placebo-controlled phase (N = 102): 37 to higher dupilumab vs. 31 in lower dupilumab. Vs. 34 in placebo<br><br>Part B (weeks 17-52): 36 week extended active treatment phase (N = 37 continued in higher dose DUP group and<br>N = 29 who continued in lower dosing DUP group).<br><br>Part C: 108 week open label extension (results have not been released yet)<br><br>Study Objective: to compare dupilumab vs placebo in patients 1=11 yrs of age with active EoE	Primary Endpoints:<br>-Histologic remission at week 16 (peak EOS count of ≤6/hpf or ≤20/square mm<br><br>Secondary Endpoints (for full list please refer to supplemental material):<br>-Peak EOS count of < 15/hpf<br>-Change from baseline in the peak EOS count<br>-Absolute change from baseline in the grade score and the EoE-HSS<br>-Absolute change from baseline in the total EREFS<br>-Change from baseline in proportion of days with at least one sign (i.e., a symptom observed by the caregiver) of EoE according to the PESQ-C.<br><br>Safety: adverse events	Part A<br>-100% of patients assigned to part A continued the higher dosing of dupilumab in part B<br>-N = 29 (94%) from part A continued lower exposure regimen during part B (weeks 17-52)<br><br>Part B<br>-Placebo group: N=18 (53%) who switched to a higher dose of dupilumab and N = 14 (41%) who switched to a lower exposure dupilumab<br><br>Peak EOS count at week 16 (histologic remission): each dupilumab dosing group was compared to the % of histologic remission in the placebo group<br>-Higher dupilumab group (68%, N = 25/37) compared to 3% of patients in the placebo group (N=1/34); 95% CI, 48-81; p < 0.001) achieved histologic remission<br>-Lower dupilumab group (58%, N=18/31)) compared to 3% of patients in the placebo group (N=1/34), (95% CI, 37-73; p < 0.001) achieved histologic remission<br>-Overall improvements in histologic measures between baseline and week 52 were similar when compared to baseline and week 16<br><br>Select Secondary Endpoints (please refer to primary article for additional secondary endpoint results):<br>-Peak EOS count < 15/hpf: 84% (N=31/37) in higher dupilumab group<br>-% change in EoE-HSS score from baseline: statistically significant when compared high dose DUP to placebo<br><br>Safety: majority classified as mild-moderate in severity<br>Part A: higher rate of COVID, injection site discomfort, headache was at least 10%	**DUP High Dose:**<br>≥ 5kg to < 15 kg: 100mg subq q 2 weeks (Parts A and B)<br><br>≥ 5kg to < 15 kg: 200mg subq q3 weeks (Part C)<br><br>≥ 15kg to < 30kg: 200mg subq q2 weeks<br><br>≥ 30kg to < 60kg (Parts A and B): 300mg subq q2 weeks OR ≥ 30kg to < 40kg (Part C only)<br><br>**DUP Low Dose:**<br>≥ 5kg to < 15kg: 200mg subq q4 weeks<br><br>≥15kg to < 30kg: 300mg subq q4 weeks<br><br>≥30kg to < 60kg: 200mg subq q2 weeks<br><br>≥ 60kg: 300mg subq q2 weeks (Part B only)

Study and Design	Population and Study Objectives	Endpoints/Outcomes	Results	Final Assessment and Pertinent Commentary
			points higher in patients who received high and low dose DUP compared to placebo.<br>Part B: Safety profile reported to be similar as in part A. In no cases in which DUP was discontinued did investigators think this was related to the study drug itself.<br><br>-<u>Serious ADRs</u> reported in 3 patients who received DUP in Part A of the study and 6 patients in Part B; Part A: one patient in Part A and placebo who had fear of injections and a drug hypersensitivity was reported with higher dose DUP in Part B.	
Nguyen N et al., 2023 [44]<br><br>**Dupilumab**<br>*Pediatric study*<br><br>**<u>FDA Approval:</u>** Dupilumab is FDA approved for treatment of EoE in children ≥ 12 years of age	<u>Study objective</u>: to assess data related to prescribing practices from both physician and patient/family perspective through retrospective chart review<br><br>Patients needed to be between 12-18 yrs of age<br><br>N= 42 who completed the MD prong of the study and N=15 for the patient portion of the study	Nonresponse to topical steroids: defined as ≥ 15 EOS/hpf despite the use of steroids<br><br>All patients were required to complete the SRBAI to assess adherence	From MD perspective (N=42): main reasons for requiring dupilumab was failure to respond to topical steroids and nonadherence.<br>-98% of patients had previously used TCS<br><br>Reported steroid adverse effects: adrenal insufficiency (17%), recurrent thrush (2%)<br><br>Dosing: 300mg qweek (90% of patients) and 300mg every other week (10% of patients)<br><br>52% of patients were prescribed dupilumab for more than one reason<br><br>From patient perspective (N = 15); 12 patients had multiple atopic comorbidities (IgE mediated food allergies and asthma). Primary	Conducted at Children's Hospital Colorado<br><br>Dupilumab is often a challenge to get approved by insurances (prior authorization frequently required; in this study 98% of cases)<br><br>Discrepancy found between reasoning of needing dupliumab when comparing MD and patient perspective: from the MD perspective, primary reasons were lack of response to topical steroids, (52%), non-adherence (28%), and adverse effects to steroids (10%). From a patient perspective, primary reasons for therapy included lack of response to topical steroids (27%), non-adherence (27%), concern related to steroid adverse effects (7%), and use for treating multiple atopic diseases (33%).
Dellon et al., 2022 [45]<br><br><br><br>**Dupilumab**<br>*Pediatric and Adult study*	Three part phase 3 trial<br><br>Patients ≥12 yrs with:<br>-Peak EOS count of ≥ 15/hpf despite high dose PPI therapy for 8 weeks<br>-≥ score of 10 on the DSQ	<u>Primary endpoints for Parts A and B</u>:<br>-Histologic remission at week 24 (peak EOS of ≤6/hpf)<br>-Absolute change in DSQ score from baseline<br><br><u>Secondary Endpoints</u> (refer to primary article for specific results related to these endpoints):-	<u>Part A:</u> histologic remission achieved in 25/42 (60%) in 300mg DUP weekly and 2/39 (5%) in placebo; p <0.001<br>-Patients with <15 EOS/hpf<br><br><u>Part B</u>: histologic remission achieved in 47/80 (59%) on 300mg DUP weekly, 49/81 (60%) on 300mg DUP q2 weeks, and 5/79 (6%) on placebo. Difference between weekly DUP and placebo was 54	Patients on PPIs prior to the study period starting were permitted to continue. New PPI initiation during the study was prohibited. No changes to diet allowed.<br><br>Patients were not permitted to use swallowed steroids within 8 weeks of the treatment period

Study and Design	Population and Study Objectives	Endpoints/Outcomes	Results	Final Assessment and Pertinent Commentary
	N = 81 in part A (42 in the 300mg DUP weekly and 39 in the placebo)<br><br>N = 240 (80 in 300mg DUP weekly, 81 in the 300mg DUP q2weeks, 79 in placebo group)<br><br>N = 40 who rec'd 300mg weekly DUP from Part A continued same dosing in Part C, N=37 who rec'd placebo in Part A where changed to qweek DUP 300mg for Part C)<br><br>Part A: 300mg DUP weekly vs. placebo<br><br>Part B: 300mg DUP weekly OR q2 weeks OR weekly placebo (for blinding pts were on the q2 week regimen were also receiving placebo q2 weeks alternative to keep blinding accurate)<br><br>Part C (for eligible patients from parts A and B of the study): those from Part A were given DUP 300mg weekly to week 52; Part C with patients from Part B is ongoing	-% change from baseline in peak EOS count<br>-change in EoE-HSS scoring, EREFS scoring, DSQ score, EoE-IQ score, and EoE-SQ score	percentage points, p < 0.001; difference between q2 week DUP and placebo was 56 percentage points (p value was not significant)<br><br>DSQ Score at week 24:<br>-Part A: on 300mg DUP q week: difference in DSQ from baseline was-12.32 when compared to placebo (p<0.001)<br>-Part B: on 300mg DUP weekly difference in score from baseline was -9.92 when compared to placebo (p<0.001)<br>-Part B: on 300mg DUP q 2weeks compared to placebo the difference in score was not significant (p=0.84)<br><br>ADRs: serious ADRs reported in 9 patients (7 on weekly DUP, 1 on q2 week DUP, 1 in placebo). 1 patient in the Part A-C group who received placebo in Pat A and weekly DUP in part C of the study	DUP given as a 300mg dose on a weekly schedule alleviated dysphagia symptoms (via DSQ score) and improved histologic outcomes when compared to placebo. DUP 300mg q week vs. 300mg q2 weeks gave similar benefit for histologic remission, but q2 week DUP vs. placebo was not significantly different
Hirano et al., 2019 [47]<br><br>**Cendakimab**<br>*Adult study*	Phase 2 trial:<br>Patients at least 18 yrs of age<br>-Symptoms of dysphagia for at least 4 days over the previous 2 week period<br>-Histological evidence of EoE based on peak EOS count of > 15/hpf at baseline	Efficacy:<br>Primary outcome: change in EOS/hpf in the 5 hpfs with highest degree of inflammation during biopsy and change in endoscopy results from baseline<br><br>Secondary outcome: DSD scoring (determined by entries in a symptom diary that was completed by each patient). Entries were done	Placebo: (16/32, 94%, completed the 16 week period; 2 patients withdrew consent).<br><br>Group 1 (180mg): 28/31, 87%, completed the 16 week period; 1 patient discontinued due to an adverse event (light headedness), 2 withdrew consent, 2 withdrew for other reasons.<br><br>Group 2 (360mg): 30/34, 88%, completed the 16 week period; 3 patients withdrew	Study was conducted across 3 countries<br><br>To be included in the study, patients had to have previously trialed PPI therapy. Patients may have previously trialed topical steroids which was noted by the investigator.<br><br>Study was not powered to determine any differences in dysphagia scoring

Study and Design	Population and Study Objectives	Endpoints/Outcomes	Results	Final Assessment and Pertinent Commentary
	N= 99; randomly assigned to one of three groups; Group 1 (N=31): 180mg, Group 2 (N=34): 360mg, Group 3 (N=34): Placebo<br><br><u>Study duration</u>: 16 weeks during the double blind period; patients were evaluated on day 1, followed by weeks 2, 4, 8, 12, and 16.	daily for at least 2 weeks consecutively during the screening period (this was 4 weeks before the study began). Also required to report daily symptoms from first day of the study to end of week 16.<br>-Other secondary outcomes included reduction in EREFS score, EESAI PRO score, peak EOS count, patient and global assessment score, change in EOE symptoms, and change in histological grade and stage.<br><br><u>Safety</u>: adverse events	due to flu-like symptoms and worsening EoE symptoms; 1 withdrew consent.<br><br><u>Primary Outcome</u>: reductions in EOS count from baseline<br><br><u>Placebo</u>: 4.42 +/- 59.94<br><u>Group 1 (180mg):</u> 94.76 +/- 67.27<br><u>Group 2 (360mg):</u> 99.9 +/- 79.53 EOS/hpf<br><br>Changes in EOS/hpf between either dose and placebo were statistically significant: $p < 0.001$ for mean EOS counts<br><br><u>Peak EOS counts</u>: 50% of patients receiving 180mg and 360mg had <15 peak EOS/hpf vs. placebo which was 0%.<br>-25% of patients in the 180mg ($p=0.0027$) and 20% of patients in the 360mg group had <6 peak EOS/hpf ($p < 0.0079$).<br><u>Select secondary outcomes</u>: treatment emergent adverse effects were similar with treatment groups and placebo (overall low)<br><br><u>EREFS (endoscopic scoring system):</u> reductions in scoring were statistically significant when comparing 180mg and 360mg to placebo ($p < 0.001$).<br><br><u>EOEHSS (histological scoring):</u> results were improved in either treatment groups vs. placebo ($p<0.001$ for both comparisons)	between groups, but results trended to show preference for the 360mg dosing scheme.<br><br>Authors could not confirm long-term effects of the drug due to the 16 week duration (longer term studies needed)<br><br>Approximately 50% of patients enrolled in the study were classified as being refractory to steroids upon randomization.<br><br>For additional information pertaining to secondary outcomes, please refer to the phase 2 study. For the purposes of this review, primary outcomes of change in EOS counts and secondary outcomes of adverse events and histologic/endoscopic staging were the primary focus of assessment.<br><br>More patients reported ADRs with the 360mg dosing compared to the 180mg or placebo groups.
Dellon ES et al., 2021 [48]<br><br>**Cendakimab**<br>*Adult study*<br><br>*LTE of the phase 2 study conducted by Hirano et al*	N = 66 (number of patients who completed the phase 2 study and enrolled into the LTE (from 16 to 52 weeks).<br><br>N=86 patients enrolled in the LTE; from this group, 20 patients did not complete the full LTE study period for the following reasons:<br>-withdrawal of consent (N=7)<br>-noncompliance (N=3)	<u>Primary efficacy outcome</u>: measured by EOS/hpf from 5 most inflamed hpfs from biopsies.<br><br><u>Secondary outcomes</u>: DSD scores, EREFS, EoEHSS, ESSAI<br>-Histologic and endoscopic outcomes were measured at weeks 12, 24, and 52. of the LTE study period	<u>Peak EOS/hpf of < 15</u>: this increased in all 3 groups from week 12 of the LTE to week 52.<br>Placebo: 28.6% to 57.1%<br>Group 1 (180mg): 53.6% to 73.9%<br>Group 2 (360mg): 44.4% to 59.1%<br><br><u>EREFS, EoEHSS: (change from baseline to week 52):</u> results are stratified based on the patient groups that were originally assigned during the double-blind trial: no significant difference noted between patients who received active drug (either dose) when compared to placebo.	To enter the LTE, participants could not have experienced any ADRs and must have had at least 80% drug compliance during the 16 week phase 2 study.<br><br>Patient in the LTE had to have received at least 1 dose of the study drug to be included in the analysis.<br><br>Results of this study were presented based on the originally assigned group from the double blind, placebo controlled trial (placebo, 180mg, or

Study and Design	Population and Study Objectives	Endpoints/Outcomes	Results	Final Assessment and Pertinent Commentary
	-investigator decision (N=1)<br>-other reasons (N=2)<br>-adverse events (N=6)<br><br>All patients received 360mg/week for dosing of cendakimab		Patients treated with either cendakimab dose maintained clinical response until week 52 of the study. Those patients who were given placebo during the double blinded portion of the study saw clinical response by week 12 of the LTE (once given active drug of 360mg/week)<br><br>Patients who were randomized to the 180mg dosing in the DB trial, did not see significant changes in clinical response during the LTE when given the higher 360mg dosing.<br><br>Most common ADRs reported: upper respiratory tract infection and nasopharyngitis	360mg). All patients enrolled in the LTE phase of this study were given 360mg dosing.<br><br>The LTE phase was not blinded.<br><br>No deaths occurred during the LTE study period<br><br>20/86 enrolled patients did not complete full 52 week study due to the following reasons: investigator decision (N=1), pregnancy (N=1), consent withdrawal (N=7), adverse events (N=6), non-compliance (N=3), other (N=2)

EOS: eosinophil count; hpf: high power field; DUP: dupilumab; DSQ: dysphagia symptom questionnaire; EoE-HSS: eosinophilic esophagitis histology scoring system; EREFS: edema, rings, exudates, furrows and strictures; EoE-IQ: eosinophilic esophagitis impact questionnaire; EoE-SQ: eosinophilic esophagitis symptom questionnaire; EGID: eosinophilic gastrointestinal diseases; TCS: swallowed topical corticosteroids; SRBAI: self-report behavioral automaticity index; ADA: anti-drug antibody; DSD: dysphagia clinical symptom frequency and severity score; EESAI PRO: eosinophilic esophagitis activity index; ADR: adverse drug reaction; LTE: long term extension; EREFS: EoE endoscopic reference score; DB: double blind; PES-QC: Pediatric Eosinophilic Esophagitis Sign/Symptom Questionnaire–Caregiver

Table S2. IL-5 Antagonists-Mepolizumab, Reslizumab, and Benralizumab for EoE-Published Data (Pediatric and Adult)

Study and Design	Population and Study Objective	Endpoints/Outcomes	Results	Final Assessment and Pertinent Commentary
Assa'ad A et al., 2011 [50,64]<br><br>International, multicenter, double-blind, randomized, prospective study<br><br>EoE defined as: baseline peak count of esophageal intraepithelial EOS of ≥ 20 in at least 1 hpf<br><br>**Mepolizumab**<br>*Pediatric study*	N = 57* (included two age ranges across 3 treatment groups): 2-7 years and 8-17 years<br><br>Group 1 (N=19): 0.55mg/kg/dose IV x 3 infusions (every 4 weeks)<br><br>Group 2 (N=20): 2.5mg/kg/dose IV x 3 infusions (every 4 weeks)<br><br>Group 3 (N=20): 10mg/kg/dose IV x 3 infusions (every 4 weeks)	12 week treatment period with 12 week follow up and 10 week long-term follow up period<br><br>Primary endpoints: proportion of patients who achieved a peak esophageal intraepithelial EOS count of < 5 per hpf at 12 weeks (per biopsy)<br><br>Secondary endpoints: change in peak and mean intraepithelial EOS counts	Peak intraepithelial EOS count of < 5 per hpf: achieved in 5/57 (8.8%) of children<br>-4 children were in the 8-17 yr age group and 1 was in the 2-7 year age group.<br>No reported differences in response when comparing across dosing schemes<br><br>Change in peak EOS count<br>-Week 12: EOS count decreased from 122.5 ± 8.78 per hpf at baseline to 40.2 ± 5.17 per hpf ($p < 0.01$)<br>-Largest decrease noted in the 2.5mg/kg group<br>-Week 24: EOS count increased to 81.7± 7.1 per hp but was still lower than baseline ($p = 0.0002$)<br>-Largest decrease noted for the 10mg/kg group<br><br>Change in mean EOS count:	Mepolizumab was successful in decreasing esophageal EOS in children with EoE.<br><br>Dosing did not seem to affect overall response rate

Study and Design	Population and Study Objective	Endpoints/Outcomes	Results	Final Assessment and Pertinent Commentary
	Study objective: to determine if anti-IL-5 antibody therapy can safely reduce the number of esophageal intraepithelial EOS in children with the EoE diagnosis		-Week 12: EOS count decreased from 39.1 ± 3.63 per hpf at baseline to 9.3 ± 1.25 per hpf ($p < 0.01$)<br>-Largest decrease noted in the 10mg/kg group<br>Week 24: EOS count increased to 23 ± 2.71 per hpf, but was still lower than baseline ($p<0.01$)	
Dellon E et al., 2023 [35,64]<br><br>Multicenter, randomized, double-blind placebo-controlled trial<br><br>**Mepolizumab**<br>*Pediatric and adult study*	N = 64/66 (97%) who completed month 3, 56/66 (85%) who completed month 6<br><br>Part 1: patients 16-75 yrs of age with EoE and dysphagia were randomized to 3 months of mepolizumab 300mg q month or placebo<br><br>Part 2: patients who were initially randomized to the mepolizumab group continued for 3 additional months; placebo patients from part 1 started 100mg of mepolizumab monthly; outcomes assessed at 6 months<br><br>Study objective: to determine if mepolizumab was more effective than placebo or decrease EOS count and improving dysphagia symptoms in adolescent and adult patients with EoE	Primary outcome: change in EEsAI from baseline to month 3 of therapy<br><br>Secondary outcomes: histological, endoscopic, and safety	Mean change in EEsAI at month 3: decrease of 15.4 ± 18.1 in mepo group vs. 8.3 ± 18 in placebo group ($p = 0.14$)<br>Mean change in EEsAI at month 6: decrease of 18.3 ± 18.1 for the mepolizumab group vs. a decrease of 18.6 ± for the placebo/mepo group ($p = 0.85$)<br><br>Peak EOS count: decreased from 113 ± 77 to 36 ± 43 in the mepo group vs. an increase from 146 ± 94 to 160 ± 133 in the placebo group ($p<0.01$)<br><br>Secondary Endpoints:<br>Histological response seen at month 3: mepo group achieved histological response of < 15 eso/hpf in 42% of patients and response of ≤ 6 eos/hpf in 34% of patients vs. 3% in the placebo group for both < 15 and ≤6 EOS/hpf in the placebo group ($p < 0.001$, $p = 0.02$)<br><br>ADRs: most commonly reported were injection site reactions; overall well tolerated	Mepolizumab did not improve dysphagia symptoms when compared with placebo<br><br>Additional improvement was not seen past 3 months of therapy<br><br>Therapy was well tolerated with minimal adverse reactions reported
Straumann A et al., 2010 [52]<br><br>Randomized, double-blind, placebo-controlled study<br><br>**Mepolizumab**<br>*Adult study*	N=11 (five in mepo group and 6 in placebo group)<br><br>Dose = 750mg of mepo x 2 infusions (1 week apart); if no response, then dose increased to 1500mg x 2 infusions (four weeks apart)	Short term follow up: 12 weeks from last infusion<br>Long term follow up: 34 weeks from last infusion<br><br>Primary endpoint: Proportion of responders (defined as EOS <5/hpf)	N=11 patients completed the study to week 21 (3 discontinued therapy due to worsening EoE symptoms; 1 was in the mepo group and 2 were in placebo group)<br><br>No patients achieved the primary endpoint of peak EOS count of <5/hpf	Mepolizumab reduced eosinophils counts with an acceptable side effect profile<br><br>Limited improvement in EoE symptoms was seen during the study period

Study and Design	Population and Study Objective	Endpoints/Outcomes	Results	Final Assessment and Pertinent Commentary
	Placebo patients received NS infusions<br><br>Study objective: to evaluate the ability for mepolizumab to reduce peak EOS to <5 EOS/hpf assessed from histology results	Clinical symptoms were assessed by a non-validated scoring system related to esophageal related symptoms (4 item questionnaire completed before study randomization and throughout the study)	Week 4: reduction in EOS count seen in mepolizumab group was 54% vs. 5% in the placebo group (p = 0.03)<br><br>Limited improvement in symptoms was seen overall<br><br>ADRS: reported by 4 patients (2 in each group); included fatigue, nausea, vomiting, esophageal impaction, and upper respiratory tract infection	
Otani I et al., 2013 [51,64]<br><br>**Mepolizumab**<br>*Pediatric study*	43 esophageal biopsy samples were obtained from 57 patients-*this was a sub-analysis of the study conducted by Assaa'ad AH et al from 2011.*<br>Dose = monthly mepo x 3 months (0.55mg/kg/dose, 2.5mg/kg/dose, or 10mg/kg/dose), then followed by no treatment until 6 months	Response to IL-5 therapy: defined as <15 EOS/hpf<br>Researchers evaluated decrease in epithelial mast cells, IL-9+ cells, and mast cell-EOS couplets in epithelium	40% of patients responded to anti-IL-5 therapy<br><br>77% of patients had decreased mast cells<br>Responders to IL-5 therapy: mast cells decreased from 62 to 19/hpf (p<0.001) and were lower in non-responders (p<0.05).<br><br>EOS and mast cells in couplets were lower in the responder group after IL-5 therapy (p<0.001).<br><br>IL-9+ cells decreased from 102 to 71/hpf after treatment (p<0.001)	Fewer mast cells, mast cell-EOS couplets, and IL-9+ cells were seen in esophageal tissue following treatment with mepo
Markowitz et al., 2018 [36,64]<br><br>**Reslizumab**<br>*Pediatric study*	N = 12 who completed the RCT (3 patients remained on RSZ for the 9 year study period)<br><br>Study objective: to evaluate the long-term safety and efficacy of RSZ (patients from RCT were enrolled into OLE and received treatment per CU)<br><br>Drug dosing: for OLE dosing was 1-3mg/kg/dose q month (dosing could be increased up to 3mg/kg per investigator discretion over a 3.5 year duration; for CU, dosing was 2mg/kg/dose q month over a duration of 5 years	Long-term follow up during the OLE (from July 2008-January 2012) and CU (from January 2012-August 2017)<br><br>Clinical remission: defined by <5 EOS/hpf, normal endoscopy results, and resolution of EoE signs and symptoms (dysphagia, abdominal pain, heart burn, vomiting, and reflux)<br><br>Safety: ADRs were evaluated on a monthly basis, were documented during the study period<br><br>Patients were excluded upon enrollment of RCT if they were receiving topical oral steroids	Average age of study participants during RCT enrollment was 12 yrs (with a range of 7-16 yrs)<br><br>58% (7/12) of patients had furrows found on endoscopy<br><br>N=3/12 (25%) patients continued on RSZ for 9 years<br><br>Resolution of symptoms:<br>N=2 (17% of patients) had vomiting that persisted during the RCT, but difficult to know due to blinding if these patients were receiving placebo or RSZ.<br><br>No patients reported vomiting during the OLE or CU phases of the study<br><br>EOS Counts: 92% of patients achieved an EOS count of <5/hpf (p<0.001)	All patients in the study were receiving PPI therapy<br><br>Diet restriction and elimination were upheld during the study period (corn, potato, rice, soy, greens, peanut, apple, and pork were restricted; the most commonly restricted foods were peanut and soy)

Study and Design	Population and Study Objective	Endpoints/Outcomes	Results	Final Assessment and Pertinent Commentary
			Median EOS count in patients on last EGD was 2 EOS/hpf. No esophageal stricture or narrowing were reported<br><br>No serious ADRs were reported<br><br><u>ADRs</u>; N= 5 who reported at least 1 ADR; nasal congestion and cough were most common	
Spergel J et al., 2012 [53]<br><br>**Reslizumab**<br>*Pediatric study*	N=226 (all with ≥ 24 EOS/hpf at baseline)<br><br><u>Study objective:</u> to evaluate the effect of RSZ in children and adolescents with EoE<br>Study was conducted in both the US and Canada (34 sites in US, 2 in Canada)<br><br><u>Patients included if</u>:<br>-Had at least one of the following symptoms within the week before randomization rated at moderate or severe intensity: dysphagia, heartburn, abdominal pain, regurgitation, vomiting<br>-had an EGD with biopsy showing ≥ 24 EOS per hpf<br>-History of treatment with PPI +/- H2RA for a minimum of 4 weeks without resolution of symptoms OR a normal pH probe (regardless of failed PPI use)<br><br><u>Study design</u>: patients were randomly assigned in a 1:1:1:1 ratio to receive one of the following which were given every 28 days (±7 days) x 4 doses total:<br>-Placebo<br>-1mg/kg of RSZ<br>-2mg/kg of RSZ<br>-3mg/kg of RSZ	<u>Primary endpoint</u>: percent change in peak EOS count from baseline to end of treatment period<br>-EOS counts determined by EGD biopsy (4 specimens total per patient)<br><br><u>Symptom Severity</u>: evaluated by physician directed global assessment and the CHQ which was done at baseline, each dosing visit, and at end of treatment<br><br><u>Safety</u>: ADRs were evaluated	Study participants were between 5 and 18 yrs of age with EoE<br><br>Percent improvement in EOS//hp was higher in the RSZ groups compared to placebo ($p<0.001$).<br><br>No differences found in EoE global assessment scores.<br><br><u>ADRs</u>: generally the drug was well tolerated; headache and cough were most commonly reported; mild infusion reactions reported. 5 patients with serious ADRs (1 in each RSZ group and 2 in placebo group). Serious ADRs included abdominal pain, anaphylaxis, respiratory distress, syncope, viral gastroenteritis, and GI inflammation. Study investigators did not feel like that any of these were from the study medication.	RSZ reduced EOS counts in children and adolescent patients with EoE. No significant differences reported quality of life or EoE symptoms.<br><br>Improvement in symptoms was not associated with change in EOS counts<br><br>All patients were permitted to take inhaled steroids, leukotriene antagonists, cromlyn, nasal steroids, but doses had to remain stable throughout the study period. No changes to diet were recommended.<br><br>The MD symptom assessment included evaluation of dietary intake, vital signs, physical findings.

Study and Design	Population and Study Objective	Endpoints/Outcomes	Results	Final Assessment and Pertinent Commentary
Rothenberg MC et al., 2024 [54]<br><br>Benralizumab<br>*Pediatric and adult study*<br><br>MESSINA study	N = 211 patients who underwent randomization (104 in BENRAL and 107 in placebo)<br><br>Four trial periods included:<br>-2-8 week run-in period<br>-24 week double blind treatment period<br>-28 week open label BENRAL<br>-Optional OLE period<br><br>Study objective: to assess the safety and efficacy of BENRAL in pediatric and adult patients with symptomatic and histologically active EoE<br><br>Study design: phase 3, multicenter, double blind, randomized, placebo-controlled trial<br>-Patients assigned 1:1 to receive subq BENRAL 30mg or placebo q4 weeks<br>-Adult patients stratified based on whether they did or didn't use swallowed steroids at baseline<br>-Adolescent patients were randomized separately<br><br>Inclusion: patients 12-65 years of age with active EoE and ≥15 EOS/hpf	<u>Primary endpoint: Efficacy at 24 and 52 weeks.</u><br>-Week 24: histologic response defined as EOS count ≤6EOS/hpf and symptom response per change in baseline of DSQ scoring<br><br><u>Secondary endpoints:</u><br>-Change in baseline of EOE-HSS scoring<br>-Change in EREFS scoring from baseline<br>-Treatment response composite of histologic response and at least 30% improvement in DSQ score<br>-Additional secondary endpoints should be reference in the primary study (list is not inclusive)	Week 24: 207 (97.6%) patients completed the double blind treatment period<br><br>N = 205 (97.2%) who completed the 28 week open label portion of the study<br><br>N=89 (42%) completed the 52 week study period<br><br>At the start of week 24, 105 patients changed from placebo to BENRAL<br><br>Histologic response at week 24 was reported at 87.4% in the BENRAL group vs. 6.5% in placebo ($p<0.001$)<br><br>Change in DSQ score was similar between groups ($p=0.18$)<br><br>Histologic response at week 52 was similar to what was reported at 24 weeks: BENRAL 83% and 89% in patients who changed from placebo to BENRAL.<br><br>No significant difference was found for EoE-HSS scoring or EREFs between groups<br><br>Safety: ADRs: most frequently were COVID-19 (12.6% in BENRAL group vs. 12.1% in placebo) and pharyngitis (7.8% in BENRAL vs. 5.6% in placebo). Two serious ADRS reported in BENRAL group vs. 1 in placebo (not thought to be related to drug therapy)	BENRAL decreased EOS counts (histologic response) at both weeks 24 and 52 but did not impact symptoms when compared to placebo.<br><br>Patients included in the study were permitted to continue on other EoE medications as long as they were stable on these medicines for at least 4 weeks prior to the run-in period.<br><br>Trial sponsor: AstraZeneca

Hpf: high power field; EOS: eosinophil count; EEsAI: EoE symptom activity index; ADR: adverse drug reaction; mepo: mepolizumab; OLE: open label extension; RCT: randomized control trial; CU: compassionate use; RSZ: reslizumab; PPI: proton pump inhibitor; EoE: eosinophilic esophagitis; H2RA: histamine 2 receptor antagonist; *there were 59 children enrolled in the study, of which 57 had 12 biopsy samples available; CHQ: children's health questionnaire; EOS: eosinophils; GERD: gastroesophageal reflux disease; IBD: inflammatory bowel disease; BENRAL: benralizumab; DSQ: dysphagia symptom questionnaire; EoE-HSS: eosinophilic esophagitis histology scoring system; EREFS: eosinophilic esophagitis endoscopic reference score

Table S3. Investigational Therapies (Pediatric and Adult)

Medication Class and Drug Class	Population and Study Objectives	Results, Final Assessment, and Pertinent Commentary
Anti-Siglec-8<br><br>Dellon E. et al., 2022 (KRYPTOS) [58]<br><br>Randomized, Double-Blind, Placebo-Controlled Phase 2/3 Clinical Trial<br><br>**Lirentelimab**<br>*Pediatric and adult study*	Primary endpoints:<br>Histological response at week 24 (proportion of patients who achieved peak EOS decrease to ≤ 6EOS/hpf<br><br>Change in DSQ score from baseline to week 24<br><br>Inclusion: patients at least 12 years of age with dysphagia and ≥ 15 EOS/hpf on esophageal biopsy; N = 276<br><br>Randomization:<br>Group 1: High dose LIR: 1mg/kg/dose x 1, followed by 3mg/kg/dose x 5 doses<br><br>Group 2: Low Dose LIR: 1mg/kg x 6 doses (monthly)<br><br>Group 3: placebo (monthly x 6 doses)	Primary endpoint (histologic response): overall cohort (N =276) achieved in 88% of patients in the high dose group and 92% in the low dose group compared to 11% in the placebo group (p<0.0001).<br><br>Adolescent patients specifically (N=51) between 12 and 17 years of age, 94% in the high dose and low dose groups met the primary endpoint when compared to placebo (6%)<br><br>Change in DSQ Score: p = 0.237 for entire cohort<br>High dose: change in 17.4 points<br>Low dose: change in 11.9 points<br>Placebo: change in 14.6 points<br><br>Change in DSQ Score for Adolescent Patients: p = 0.1316<br>High dose: change in 18.4 points<br>Low dose: change in 16.4 points<br>Placebo: change in 8.9 points<br><br>ADRs: infusion related reactions and headache<br>Infusion reaction: high dose was 38.5%, low dose 25.8%, and placebo 12%<br>Headache: High dose was 6.6%, low dose 8.6%, and placebo 6.5%<br><br>Serious ADRs: reported in 3 patients (2 in high dose group and 1 in placebo): study authors did not specify what ADRs were seen
Anti-TSLP<br><br>AstraZeneca 2024 (CROSSING- NCT05583227) [60]<br><br>Randomized, Double-Blind, Parallel-Group, Placebo-Controlled Phase 3 Efficacy and Safety Study<br><br>*granted orphan drug designation from the FDA to treat EoE in 2021*<br><br>**Tezepelumab**<br>*Pediatric and adult study*	Inclusion: 12-80 years of age and at least 40kg<br>-Established EoE diagnosis with confirmed biopsy<br><br>Exclusion: other GI disorders such as H pylori, IBD, use of feeding tube, hypereosinophilic syndrome, esophageal dilation performed within 8 weeks prior to study screening<br><br>52 week randomized study: patients have the option of participating in the extension period for an additional 24 weeks<br>-Patients who do not participate in extension, will have a 12 wee kof period treatment follow up<br>-Expected N =360 randomized in a 1:1:1 ratio to 3 separate treatment arms:<br>1) High dose TEZ<br>2) Low dose TEZ<br>3) Placebo<br><br>Primary Endpoints:<br>-Histologic response defined by ≤6 EOS/hpf<br>-DSQ response	**Anticipated study completion: January 2027**<br><br>May be on concomitant PPI therapy

<table>
<tr><th>Medication Class and Drug Class</th><th>Population and Study Objectives</th><th>Results, Final Assessment, and Pertinent Commentary</th></tr>
<tr><td>(Alpha-1 Trypsin Inhibitor) [61,64]

Phase 2, Open-Label Trial

Zemaira
Adult study</td><td><u>Inclusion:</u> patients 18-70 years of age
-Histologically active EoE with peak EOS count of 15/hpf
-History of moderate to severe symptoms
-History of at least 8 weeks of SOC

<u>Exclusion</u>: current H. pylori infection, Barrett’s esophagus, cancer, receiving ZEM for other indications (note this is not an all-inclusive list of criteria)

<u>Treatment</u>: weekly infusions of ZEM at 120mg/kg/dose IV x 12 infusions

<u>Primary Endpoint:</u>
-Absolute change in A1AT esophageal concentrations in patients receiving ZEM to 24 hours post last infusion at 12 weeks
-Number of ADRs related to the study drug at 24 weeks

<u>Secondary Endpoints:</u>
-Absolute change from baseline serine protease activity to end of treatment visit at 12 weeks</td><td>Anticipated study completion: December 2025

SOC = PPI, topical steroids</td></tr>
<tr><td>S1P Modulator

Dellon et al., 2023 (VOYAGE) [63,65]

Phase 2, Randomized, Double-Blind, Placebo-Controlled Study

Etrasimod
Adult study</td><td><u>Inclusion</u>: patients 18-65 years old
-Diagnosis of EoE
-Peak EOS ≥15/hpf
-Minimum of 2 episodes of dysphagia within 2 weeks of study enrollment

<u>Exclusion:</u> treatment with swallowed topical steroids within 8 weeks prior to study screening
-Change in PPI therapy within 8 weeks prior to screening
-Esophageal dilation: within 12 weeks prior to study screening

<u>24 week placebo controlled study: N = 108</u>
1) ETRA 2mg daily (N = 41)
2) ETRA 1mg daily (N=39)
3) Placebo daily (N = 28)
<u>28 week extension period:</u>
1) From the 41 patients in the 24 week study, 30 continued into the extension phase
2) From 39 patients in the 24 week study, 31 continued into the extension phase
3) From the 28 patients in the 24 weeks study, 12 were randomized to 2mg ETRA and 12 to the 1mg dosing group

<u>Primary Endpoint:</u> Percent change in peak EOS count from baseline to week 16

<u>Secondary Endpoints:</u>
-Proportion of patients who achieved peak EOS < 15 and ≤6/hpf</td><td><u>ETRA 2mg:</u> 46.1% decrease from baseline at week 16 (p=0.0103)

<u>ETRA 1mg</u>: 32.5% decrease from baseline at week 16 (p =0.2861)

<u>Placebo:</u> 7.8% change in baseline

<u>Pertinent Secondary Endpoints:</u>
ETRA 2mg: Peak EOS (p <0.0001), EoE-HSS (p<0.0001), PGIS (p=0.0121)
ETRA 1mg: Peak EOS (<0.0022), EoE-HSS (p=0.001)

<u>Final conclusion:</u> ETRA 2mg met the primary histologic endpoint, as well as EoE-HSS and PGIS</td></tr>
</table>

Medication Class and Drug Class	Population and Study Objectives	Results, Final Assessment, and Pertinent Commentary
	-Absolute change from baseline in EoE-HSS<br>-Absolute change from baseline EREFS, PGIS, and DSQ score<br><br>Safety: treatment emergent adverse events	

EOS: eosinophil; hpf: high power field; DSQ: dysphagia symptom questionnaire; LIR: lirentelimab, AK002; ADR: adverse drug reaction; IBD: inflammatory bowel disease; TEZ: Tezepelumab; S1P: selective sphingosine 1-phosphate receptor modulator; SOC: standard of care; A1AT: alpha 1-anti-trypsin; ZEM: Zemaira; PPI: proton pump inhibitor; EoE-HSS: eosinophilic esophagitis histology scoring system; EREFS: EoE-endoscopic reference score; PGIS: patient global impression of severity; DSQ: dysphagia symptom questionnaire; ETRA: etrasimod;.

Table S4. Anti-IgE-Omalizumab for EoE-Published Data (Pediatric and Adult)

Study and Design	Population and Study Objectives	Results
Arasi S et al., 2016 [39,66]<br><br>*Pediatric study*	Case report with a single patient<br><br>13 year old boy with severe asthma and EoE which was not responsive to a strict elimination diet<br><br>At 11.5 years of age treatment was initiated with OMAL 375mg subq q2 weeks based on IgE serum levels and weight	After 3 months of treatment, was in clinical remission but not histologic remission for EoE<br><br>At 7 months of treatment had exacerbation of GI symptoms which required initiation of topical steroids<br><br>OMAL did not produce persistent histologic or clinical improvement with EoE
Clayton F et al., 2014 [56]<br><br>Prospective, randomized, placebo-controlled trial<br><br>*Adult study*	Study objective: to determine if EoE is an IgE mediated<br><br>OMAL (N = 16)<br>Placebo (N = 14)<br><br>OMAL q2-4 weeks x 16 weeks (dosing based in participant weight and IgE serum levels)<br><br>Patients included if:<br>-At least 18 years of age<br>-Active EoE (IOS ≥ 15/hpf) while receiving max PPI therapy<br>Primary endpoints: EOS counts and clinical symptoms	No significant effects were seen in clinical symptoms or EOS content when comparing OMAL groups to placebo
Loizou D et al., 2015 [41]<br><br>Unblinded, open-label, single center study<br><br>*Pediatric and adult study*	N = 15 (age 12-75 years)<br><br>Study Objective: to evaluate the role of OMAL in esophageal tissue inflammation<br><br>Drug Dosing: calculated in a weight based fashion (mg/kg/dose) per IgE unit/ml. Dosing was either q2 weeks or q4 weeks (dependent on dosing of OMAL for allergic asthma)<br>-Total study duration 12 weeks<br><br>Patients included if:<br>-Between 12 and 75 years of age<br>-Evidence of atopy<br>-Total serum IgE between 30 and 700 IU/ml<br>-Peak EOS > 15/hpf<br>-Failure to treat symptoms with food avoidance or steroids<br><br>Primary Endpoints: histologic remission of EoE and clinical improvement	Median age: 14 years<br><br>-Average peak EOS prior to OMAL: 30.2/hpf<br><br>-60/78 doses were administered to pediatric patients (< 18 years old)<br><br>-5/15 (approx. 33%) of study participants had full histologic remission<br>-Symptom scores improved in 7/15 patients when comparing before OMAL and after OMAL therapy (p=0.018)<br>-No symptomatic improvement was seen in patients who did not experience histologic improvement

Study and Design	Population and Study Objectives	Results
	-Peak EOS < 15/hpf and endoscopic remission of disease<br>-Symptom score improvement<br><br><u>Safety</u>: ADRs	Pediatric Patients: 4/11 participants achieved full remission<br><br>-<u>ADRs:</u> no anaphylaxis, serum sickness, cardiovascular events, or malignancy reported<br><br>-No changes were made to participant diet or baseline medications during the study period
Rocha et al., 2011 [40]<br><br>*Pediatric study*	Case report with 2 patients<br><br>Patient #1: 18 year old female with active EoE: OMAL 300mg subq every 2 weeks after failing oral budesonide<br><br>Patient #2: 9 year old male with Hirschprungs and multiple food allergies and active EoE: OMAL 300mg subq every 4 weeks after not tolerating topical steroids<br><br>Primary Endpoints: histologic remission and symptom control	Patient #1: had improvement in allergy symptoms and skin and digestive manifestations. At 6 months, did not show improvement with endoscopy or histology<br><br>Patient #2: had improvement in allergic response. Endoscopy and histology remained unchanged when compared to before OMAL therapy was initiated

EoE: eosinophilic esophagitis; OMAL: omaliziumab; ADR: adverse drug reaction; PPI: proton pump inhibitor.

<u>Table S5. Anti-TNFα-Infliximab (Adult only)</u>

Drug and Study Design	Population and Study Objectives	Results
Straumann A et al., 2008 [38,18]<br><br>Infliximab<br>*Adult study*	Inclusion: 3 adult male patients with steroid refractory EoE<br>-Active EoE: dysphagia + peak EOS > 24/hpf<br><br><u>Primary Endpoint</u>: effect of infliximab on eosinophilic infiltration<br><br><u>Secondary Endpoint:</u> effect of infliximab on symptoms<br><br><u>Treatment:</u> Infliximab 5mg/kg/dose IV at weeks 0 and 2	-All patients had previously tried PPIs, topical steroids, dilations, and systemic steroids<br>-Improving symptoms in patients 1 and 3 and increasing symptoms in patient 2<br><br>Change in peak EOS count in the esophageal tissue (mean cells/hpf) four weeks after 2nd infliximab infusion:<br>-Patient 1: 102.5 at baseline and decreased to 72<br>-Patient 2: 157.1 at baseline and increased to 237.5<br>-Patient 3: 44.9 at baseline and increased to 54.2<br><br>Symptoms: were not significantly reduced<br><br>No reported ADRs<br><br>Infliximab did not result in significant improvement for 2/3 patients who were treated.

EoE: eosinophilic esophagitis; ADR: adverse drug reaction.
